# Population dynamics of threatened felids in response to forest cover change in Sumatra

**DOI:** 10.1371/journal.pone.0236144

**Published:** 2020-08-12

**Authors:** Iding A. Haidir, David W. Macdonald, Wai-Ming Wong, Muhammad I. Lubis, Matthew Linkie

**Affiliations:** 1 Kerinci Seblat National Park, Indonesian Ministry of Environment and Forestry, Jambi, Indonesia; 2 Wildlife Conservation Research Unit (WildCRU), Department of Zoology, University of Oxford, The Recanati-Kaplan Centre, Tubney, United Kingdom; 3 Panthera Foundation, New York, New York, United States of America; 4 Durrell Institute of Conservation and Ecology, University of Kent, Canterbury, United Kingdom; 5 Wildlife Conservation Society Indonesia Programme, Bogor, Indonesia; Michigan State University, UNITED STATES

## Abstract

Habitat loss caused by deforestation is a global driver of predator population declines. However, few studies have focussed on these effects for mesopredator populations, particularly the cryptic and elusive species inhabiting tropical rainforests. We conducted camera trapping from 2009–11 and 2014–16, and used occupancy modelling to understand trends of Sumatran mesopredator occupancy in response to forest loss and in the absence of threats from poaching. By comparing the two survey periods we quantify the trend of occupancy for three sympatric felid species in the tropical rainforest landscape of Kerinci Seblat National Park. Between 2000 and 2014, forest loss across four study sites ranged from 2.6% to 8.4%. Of three threatened felid species, overall occupancy by Sunda clouded leopard (*Neofelis diardi*) and Asiatic golden cat (*Catopuma temminckii*) remained stable across all four areas between the two survey periods, whilst marbled cat (*Pardofelis marmorata*) occupancy increased. In general occupancy estimates for the three species were: lower in lowland forest and increased to attain their highest values in hill forest, where they declined thereafter; increased further from the forest edge; positively correlated with distance to river, except for golden cat in the second survey where the relationship was negative; and, increased further from active deforestation, especially for clouded leopard in the second survey, but this was some 10-15km away. Our study offers fresh insights into these little known mesopredators in Sumatra and raises the practically important question of how far-reaching is the shadow of the encroachment and road development that typified this deforestation.

## Introduction

A global review of felids and other large-bodied Carnivora revealed a general pattern of population decline that was primarily caused by habitat conversion to agriculture, poaching for trade, retaliatory killing from conflict with people, and stochastic events [1]. For example, the leopard (*Panthera pardus*), one of the world’s most widely distributed felid species, has lost approximately 18–67% of its historic range in Africa, and 83–87% in Eurasia over the past 250 years [2]. Densities of jaguar (*Panthera onca*) across South America have been suppressed by human presence and their genetic diversity is threatened by habitat fragmentation, especially in critical habitat such as Brazilian Atlantic Forest [3–5]. Similarly, many felid populations in tropical Asia, such as clouded leopard (*Neofelis diardi* and *Neofelis nebulosa*) and tiger (*Panthera tigris*) in tropical Asia are declining due to inadequate protection, habitat loss, and/or the high demand for their body part [6, 7].

Monitoring trends in felid populations in tropical forests is technically challenging, because they tend to be elusive, to occur at low densities, and in dense vegetation. The effort and cost of surveying large-bodied felids has been high for tiger [8], leopard [9], and jaguar [10]. In contrast disproportionately small sums (<1% of global funding for felid conservation) have been allocated to field research and conservation of small felids, for which some 80% lack robust population assessments (J. Sanderson, unpublished data). This imbalance applies on the Indonesian island of Sumatra, where of the six resident felid species, only the Sumatran tiger (*P*. *t*. *sumatrae*) has been monitored effectively [11–16].

The smaller-bodied felid species in Sumatra include the Vulnerable Sunda clouded leopard (*Neofelis diardi*), the Endangered flat-headed cat (*Prionailurus planiceps*), the Near-Threatened Asiatic golden cat (*Pardofelis temminckii*), the Near-Threatened marbled cat (*Pardofelis marmorata*), and the Least Concern leopard cat (*Prionailurus bengalensis*). Of the limited research conducted on these species on Sumatra, only one study has established a baseline density estimate, which was for the clouded leopard [17]. Maximum entropy modelling, using presence-only data, has been used to predict the distributions of clouded leopard, Asiatic golden cat, leopard cat, and marbled cat using camera trap data [18]. That study provided baseline data for these species in southern Sumatra, where clouded leopard, golden cat and marbled cat, respectively, occurred more widely than the smallest cat, leopard cat. Haidir et al. [19] provided the first estimates of occupancy, habitat associations and niche overlap between the Sunda clouded leopard and Asiatic golden cat. A recent range-wide estimate of Sunda clouded leopard distribution suggested that this species occupied a third of the available forest blocks in Sumatra, particularly hill forest (300-800m above sea level and Dipterocarp dominated), whereas in Kalimantan they occurred at mid-elevation (600m+) protected areas [13].

Here, we assess occupancy as an indicator of population trends [20], of smaller-bodied felid species in the UNESCO World Heritage Site of Kerinci Seblat National Park, Sumatra, and the surrounding forests. We selected four study areas that have been systematically surveyed using camera traps set from 2009–2011 [21] and 2014–2016 [19]. More specifically, we use these temporal data to investigate: 1) the occupancy trends of the Sunda clouded leopard, Asiatic golden cat, and marbled cat using single-species, single season occupancy modelling; and, 2) patterns of deforestation and the accompanying response of these species to this type of habitat change.

## Materials and methods

The study was conducted in Kerinci Seblat National Park and its adjacent forest that are under State land authority. A permit to conduct the fieldwork was provided by the Indonesian Ministry of Environment and Forestry (MoEF) technical unit Kerinci Seblat National Park (KSNP) Authority for Jambi, West Sumatra, South Sumatra and Bengkulu regions, of whom the first author is an employee of KSNP, MoEF. Our field teams had a permit from the national park head office and did not therefore require written permission from the village heads, but instead had their verbal permission.

### Study area and sampling design

Our study area was the Kerinci Seblat Landscape that consists of national park forest, production forest, watershed forest, and wildlife and nature reserve forest that are designated for the protection of particular wild fauna and their natural habitats. The ~16,000 km^2^ contiguous landscape lies on the Bukit Barisan mountain range that for the Kerinci Seblat section stretches ~370 km and encompasses forested areas in bordering provinces of West Sumatra, Jambi, Bengkulu and South Sumatra. We conducted two consecutive camera trap surveys in four study areas in this landscape for the years 2009–11 and 2014–15, hereafter respectively referred to as surveys I and II. The study areas were selected because they represented the main elevation classes for the landscape: lowland (ranges from 0–300 m above sea level), hill (300–800 m asl), sub-montane (800–1900 m asl), and montane (>1900 m asl).

We deployed a combination of passive infrared camera traps: 20 units of Bushnell (Bushnell Corporation, Overland Park, KS, USA), 10 units of Highlander Photoscout and Moultrie (Moultrie^TM^, Alabaster, AL, USA) for survey I, and 160 units of Cuddeback Ambush IR (Non Typical Inc., WI, USA) and 36 units Panthera IV (Panthera Foundation, USA) particularly in Bungo, for survey II. No lures or attractants were used at any camera trap locations. Cameras were placed along the ridges and animal trails at a height of 40–60 cm above the ground and a distance of 2–3 metres from the target trail. Cameras were active 24 hr/day and set with a 5-minutes delay between exposures. Camera trap spacing ranged 1–4 km from one another. The cameras recorded time and date of each photographed animal. Every two weeks, two teams of 4–5 people visited camera locations for maintenance and data retrieval during ~100 days of camera trap operation.

### Focal species occupancies

This study targets three focal felid species: Sunda clouded leopard, Asiatic golden cat, and marbled cat. Leopard cat is present but was excluded due to the low sample size. Flat-headed cat (*Prionailurus planiceps*) is strongly associated with peat swamp forest, a habitat that is absent from our study area. To estimate species occupancy, a single species, single season occupancy model was used, based on four main assumptions: i) occupancy state is closed, where species occupancy and detection probability at all sites remained constant over a survey period but may change between surveys; ii) trap locations (sites) and replicates are spatially and temporally independent, where detecting species of interest at a site is independent of detecting the species at other sites or other time interval, iii) site and survey covariates factors that influence occupancy are quantified and incorporated in the model calculation, and iv) factors that influence detection probability are explained through incorporating site covariates and survey covariates within the analyses [22], although in this study the latter assumption is considered constant across sites [23].

Focal species detection matrices of two series of surveys were constructed where ‘1’ annotated a detection, ‘0’ a non-detection, and ‘-’ indicating a camera was not running during the time interval, where each sampling occasion (*K*) consisted of 14 trap days. The survey data used spanned 90–120 days that adhere to population closure assumption [24] and only adult individuals were included in our detection matrices.

Using the R package ‘camtrapR’ [25], camera trap data were extracted and converted from each survey into unique detection matrices (*K* = 14 days). We identified six site covariates that were potentially incorporated into the models: elevation and slope derived from SRTM [26]; distance to forest edge using global forest cover change data derived from Hansen et al. study [27]; distance to river, distance to road, and distance to village using rasterized data produced by the National Mapping and Survey Agency of Indonesia (BAKOSURTANAL, 2017); and, distance to deforestation polygons (>100 hectares/1km^2^) in each survey, obtained from BAPLAN (Indonesian Ministry of Environment and Forestry’s Planning and Mapping Centre).

We used the Euclidean distance to deforestation within a 15km radius from the outermost camera trap polygon associated with their respective surveys from the year 2010 or year 2014. We tested distance to forest edge and to deforestation, accepting these as proxies for habitat quality and degradation. Forest loss was calculated using data taken from the global forest dataset [27], which provides high resolution data on tree canopy cover at approximately 30 m resolution. Following Beaudrot et al., [28], we defined tree canopy for our tropical rainforest cover as being >75%. Deforestation was then defined as the complete conversion of a forest pixel to a non-forest pixel between survey periods. We measured forest cover and change for each study area including a 15-km buffer around the outermost camera trap locations, because deforestation can have wider reaching impacts. Using ‘gfcanalysis’, ‘rgeos’, ‘raster’, ‘rgdal’ in R software [29, 30], forest cover changes were calculated annually and clustered into two periods: i) 2005–2009 to correspond with camera trapping period I; and, ii) 2010–2014 to correspond with camera trapping period II.

We performed multicollinearity tests and, where correlated covariates were found, ensured that they were not included in the same occupancy model [31]. We used "layerStats" function in package raster [29] to compute correlation and (weighted) covariance for multi-layer raster objects in R version 3.5.3. Based on this test, we dropped variables that exhibited correlations >0.5. This resulted in a final data set consisting of scaled distance to deforestation, scaled elevation (quadratic function), scaled distance to rivers, and scaled distance to forest edge. We chose these variables because they are uncorrelated, and for comparability with earlier studies by Haidir et al. [19] that had found them to influence mammalian occupancy in the study areas [23, 32].

We performed single species single season occupancy analyses for each of the focal species for each study area and for each survey period. We then combined data from all study areas for each survey period and used study area as one of the covariates. We ranked the plausible models for small sample sizes using AICc (ΔAICc <2) and calculated model averages for each survey period, where possible, in each study area [15, 23, 33]. All calculations of model averaging, where each model was multiplied by its model weight, in order to get model rankings, were performed using ‘wiqid’ package [34].

The occupancy values derived from the model averaging procedure were used to test for significant differences of pairwise study areas over the study periods, where non-overlapping 95% confidence intervals, as a first step, indicate a significant difference. However, overlapping intervals may still be significantly different, and in such situations, a Tukey’s Honest Significant Difference (HSD) test was subsequently performed, with *P* < 0.05 taken as the significance level (i.e. Pr(>|z|)) [1]. All model calculation, covariates analyses, raster analyses and statistical tests were performed in R Studio [35].

## Results

### Focal species detection

For both surveys, 246 camera trap locations generated 21,539 trap nights (Table 1) and detections of four small-medium sized cat species: Sunda clouded leopard (n = 163), Asiatic golden cat (116), and marbled cat (55), with two leopard cat detections and no flat-headed cat detections. Clouded leopard, golden cat and marbled cat were recorded in all four study areas and during both sampling periods (S1 Table).

**Table 1:** Summary of survey effort and characteristics of the study areas over two sampling periods: 2009–11 (I) and 2014–15 (II).

Feature	Bungo		Sipurak		RKE		Ipuh	
	I	II	I	II	I	II	I	II
Main forest type	Hill-submontane		Hill-submontane		Hill-montane		Low land-hill	
Mean elevation (min-max) in meter asl	753 (389–1355)	627 (307–1116)	883 (700–1253)	811 (575–1069)	1264 (593–1985)	1198 (489–1992)	512 (145–1032)	427 (263–649)
Annual deforestation 2004–2014 in 15 km buffer (percentage forest loss)	0.60% (6.38%)		0.88% (8.43%)		0.26% (2.59%)		0.61% (5.91%)	
# camera locations	21	40	21	40	23	40	21	40
Trapping area (km2)	76.5	63.9	71.8	62.5	91.9	63.2	140.8	60.6
Trapping dates (dd/mm/yy)	13/04/10 to 16/07/10	11/06/14 to 11/11/14	24/12/09 to 26/03/10	24/11/14 to 08/04/15	28/07/10 to 27/10/10	25/04/15 to 10/08/15	28/11/10 to 03/03/11	06/09/15 to 24/12/15
# Trap nights	1886	4247	1715	3504	2021	3090	1893	3183

### Occupancy changes

**Fig 1:**
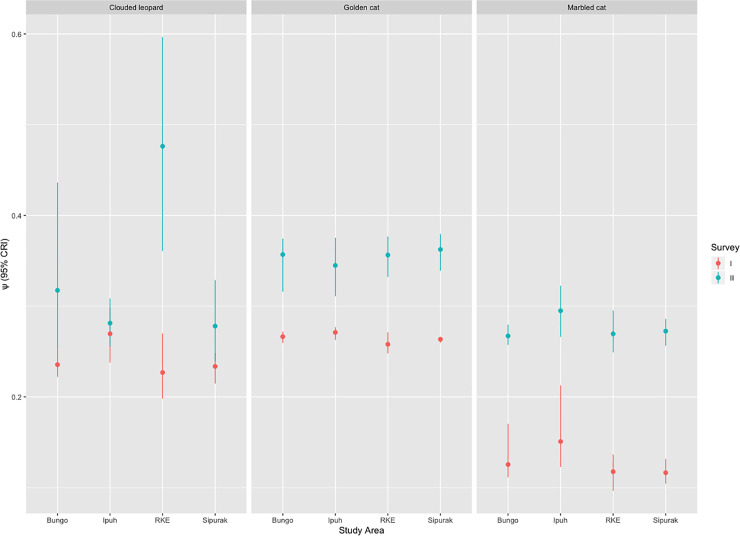
Focal species occupancy trends from survey periods I (2009–11) and II (2014–2016), points are mean of occupancy, bars indicate 95% credible interval.

The constant model occupancy ($\hat{\psi }$) for clouded leopard in all study areas combined was 0.24 in survey I and 0.33 in survey II, while for the sympatric golden cat it was 0.27 and 0.36, and for marbled cat it was 0.13 and 0.27 (S2–S4 Tables). The top-ranked model predictions $\left(\hat{\psi }\right)$(±SE) for all study areas combined over the two survey periods were: clouded leopard = 0.24 (±0.05) and 0.33 (±0.04), with respective detection probabilities ($\hat{p}$) of 0.39 and 0.22; Asiatic golden cat = 0.27 (±0.06) and 0.36 (±0.05), with respective detection probabilities of ($\hat{p}$) 0.31 and 0.14; and, marbled cat = 0.13 (±0.04) and 0.28 (±0.04) with respective detection probabilities of ($\hat{p}$) 0.31 and 0.11 (Fig 1 and S2–S4 Tables). For clouded leopard and golden cat, distance to forest edge was the single most frequently occurring covariate in the top ranked model or candidate models within two ΔAICc units of the top model. The constant model remained the top candidate model for marbled cat in both surveys (S2–S4 Tables).

**Fig 2:**
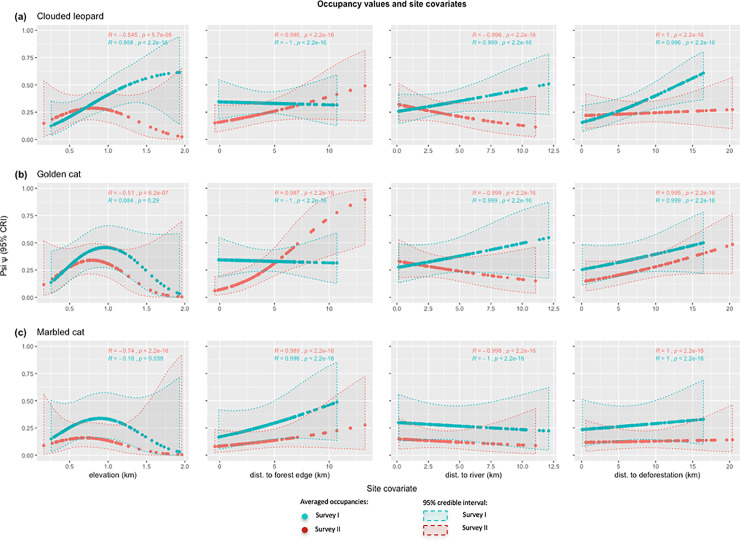
Relationships between occupancy values and covariates, where R is slope of relationships between psi and respective covariates and p is the significance value.

The general trends shared across the three cat species were: i) occupancy estimates were lower in lowland forest and increased to their highest values between 700 and 1000 m above sea level (mainly hill forest type), where they declined thereafter, except for clouded leopard in survey II; ii) occupancy increased further from the forest edge, except for clouded leopard and golden cat in survey II where there was a non-significant change, instead showing that they have slightly higher occupancy near forest edge; iii) occupancy was positively correlated with distance to river, exhibiting a linear relationship, except for golden cat in survey II where the relationship was negative; and, iv) occupancy increased further from active deforestation especially for clouded leopard in survey II (see Fig 2 for more details).

Comparing the occupancy estimates and their associated confidence intervals, Tukey’s test indicated non-significant occupancy increases (Pr(>|z|) >0.05) for clouded leopard and golden cat, whereas marbled cat had a significant increase of 77.2% (Pr(>|z|) < 0.05) from the first survey (S2 Table–S4 Table for details of changes of occupancy values).

### Deforestation patterns

**Fig 3:**
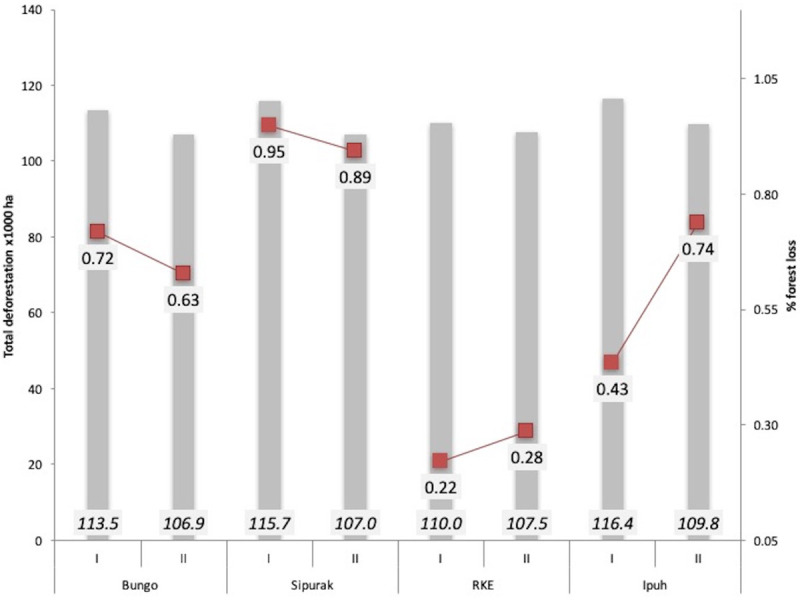
Forest cover change in four study areas over two sampling periods: 2005–2009; and, 2010–2014 (bars) and mean annual percentage of forest loss with red lines indicating the temporal trend.

From 2005–2014, our remote sensing analysis recorded forest area baselines and annual rates of forest loss in Sipurak (baseline = 115,307 ha; mean annual forest loss of 0.93%; 1037 ha lost/year), Bungo (baseline = 112,876 ha; 0.67%; 739 ha/year), Ipuh (baseline = 115,721 ha; 0.61%; 693 ha/year) and RKE (baseline = 110,038 ha; 0.28%; 304 ha/year; Fig 3, Table 2). Forest loss trends from the different study areas showed wide year-over-year fluctuations, but the overall net value of forest change across all study areas combined from 2005–2014 was -24,970 hectares, equivalent to 5.5% of the entire study areas. For the overall forest loss in the 15km buffered camera trap polygons in each study areas over two survey periods, see S2 Fig.

**Table 2:** Forest cover (in hectares) and forest loss (in hectares and as a percentage change) in four study areas prior to camera trap survey I in 2010–11 and survey II in 2014–16.

Year	Study area			
	Bungo	Sipurak	RKE	Ipuh
*Forest cover (hectares)*				
2005	112,876.2	115,307.4	109,931.2	115,721.4
2009	109,608.6	110,838.9	108,728.5	113,609.1
2010	109,182.3	110,270.4	108,506.1	113,142.2
2014	106,221.6	105,972.6	107,190.7	109,481.2
*Forest loss*, *hectares (%)*				
2005–2009 annual average	-817.9 (-0.7%)	-1,117.1 (-1.0%)	-301.7 (-0.3%)	-528.1 (-0.5%)
2005–2009 total area	-3,267.6 (-2.9%)	-4,468.5 (-3.9%)	-1,202.7 (-1.1%)	-2,112.3 (-1.8%)
2010–2014 annual average	-740.2 (-0.7%)	-1,074.4 (-1.0%)	-685.1 (-0.6%)	-1,560.1 (-1.4%)
2010–2014 total area	-2,960.7 (-2.7%)	-4,297.7 (-3.9%)	-2,740.5 (-2.5%)	-6,240.2 (-.5.5%)

Deforestation data from 2005 to 2009 were clustered as an initial deforestation rate in the first study period. Similarly, deforestation data between 2010 and 2014 were clustered for the deforestation calculation over study period II.

## Discussion

This study is an important addition to the limited body of scientific knowledge on Southeast Asia’s small-medium sized cat species, particularly from Sumatra [18, 36]. Our approach could usefully be applied widely [37]. Kerinci Seblat National Park, along with the national parks of Gunung Leuser and Bukit Barisan Selatan, form the Tropical Rainforest Heritage of Sumatra that have been placed on the UNESCO ‘in danger’ list since 2011. They must collectively demonstrate that the impact of their mitigating actions are assisting in the recovery of the priority species; we provide that evidence for the smaller bodied felids [38].

For example, occupancy studies have been used to assess the spatio-temporal distribution of herbivores in India [39], set population baselines and harvesting thresholds for fisher (*Pekania pennanti*) in the United States [40], and identify conservation priorities for multiple threatened species in production landscapes in Sumatra [15]. Nonetheless, the occupancy approach has limitations with respect to monitoring difficult to detect and habitat specialist species for which data are sparse. The two leopard cat detections, and perhaps even the 55 marbled cat detections, are a case in point. For occupancy modelling to detect significant change in population indicators may require hundreds of camera traps for species with a low detection probability [41], of which in this study we did not test how types of camera trap unit affect detection probability. An additional consideration, relevant to our sampling design, is improving camera trap placement for semi-arboreal species–a factor that may apply to both clouded leopard and marbled cat. Nonetheless, and despite recognizing these possible limitations, we were able to record >100 independent records for both clouded leopard and golden cat. Our repeat surveys were conducted at times of year that overlapped by >60%. It is possible that more modern cameras, even of the same brand [42] may have increased detectability in the second survey.

A camera trap study conducted in the periphery of three Sumatran national parks—Gunung Leuser, Kerinci Seblat, and Bukit Barisan Selatan—found ongoing pressure from forest loss and habitat degradation outside of the protected areas might explain the higher, perhaps even inflated, tiger densities inside the park [43]. In Sabah, on the neighbouring island of Borneo, the carnivore community had ~28% higher relative abundance in previously logged forest compared to old growth forest [44]. These findings indicate the pernicious threat posed by active deforestation but also that populations of certain carnivore species can recover in degraded forests, if the threats are removed, in turn underscoring their conservation value. From our second survey period, clouded leopard and golden cat maintained higher occupancies in areas closer to the forest edge, nearby formerly selectively-logged or degraded forests. We speculate that prey, such as murid rodents, may be more abundant in these peripheral areas [44].

Our forest cover analyses showed that the rate of deforestation fluctuated over the observed periods. It increased between 2005 and 2009 and then from 2010 onwards slowed down, following a nationwide pattern for Indonesia [45]. In our study area, we detected a subtle relationship between forest cover change and the presence of monitoring personnel: rates of forest loss were lower when research staff were active. We recorded that in 2010, forest loss in all study areas combined was 35–45% lower than the mean value, and forest loss in Bungo decreased by 52%. These periods coincided with the activity of monitoring teams in the vicinity from December 2009 to March 2011 [21]. This is in accord with a study on 98 protected areas in 15 tropical African countries that found non-protection activities such as research and tourism, when conducted on a regular basis, could reduce illegal activities in core areas, and hence improve habitat quality and support the recovery of wildlife populations [46].

Despite the levels of forest habitat loss we recorded, there was no evidence of reduced occupancy. One consideration is that the deforestation occurred some 10–15 km away from our camera-trapping grids, perhaps too far for a discernible effect to be felt by our study animals. Studies of a similar mesocarnivore, the African golden cat, reported that increased cultivation adjoining protected areas was associated with a six-fold density decrease on farmland compared to pristine forest [47]. In Madagascar, an increase in habitat degradation suppressed native carnivore occupancy, yet concentrated it in some areas, and with decreased encounter rates [48].

Considering our results more widely, assigning strict protection zones in the more accessible and therefore at-risk areas of the park with regular presence of law enforcement and monitoring personnel should reduce illegal activities [49]. Additionally, from Kerinci Seblat NP management point of view, an IUCN monitoring mission recommendation to the management authority was to implement an emergency action plan to withdraw the national park from the Tropical Rainforest Heritage Sites in danger list [38]. Key to this, as with other protected areas in Indonesia, is progressing from securing smaller core sites and expanding law enforcement and monitoring efforts to the wider landscape for ensuring the population viability of species such as golden cat, clouded leopard, marbled cat and other threatened species [14].

## Supporting information

S1 Data (ZIP)

S1 Table Small-medium sized wild cat detection encounter rate (#independent photo/100 trap days) and standard deviations in parentheses from the two survey periods and four study areas. (DOCX)

S2 Table Top occupancy models of clouded leopard from two periods of all study areas combined, of survey I and II. (DOCX)

S3 Table Top occupancy models of golden cat from two periods of all study areas combined, of survey I and II. (DOCX)

S4 Table Top occupancy models of marbled cat from two periods of all study areas combined, of survey I and II. (DOCX)

S1 Fig Map of study areas across Kerinci Seblat National Park and its adjacent forest in west-central Sumatra. *RKE: Renah Kayu Embun.
(TIFF)

S2 Fig Forest cover changes over study period. Solid thin black line is KSNP border, bold solid black line is camera trap polygon of survey I, dashed bold black line is survey in period II, and thin dashed black line is 15 km buffer from the outer most camera trap polygon. Black shaded polygons are deforested area, grey polygons are the existing non-forested area, and light greens are: cultivated, plantation and settlement, white polygon is water body. Forest cover changes shown in this figure are a year ahead before the surveys were carried out.
(TIFF)
