# Supplementary material for: Population dynamics of threatened felids in response to forest cover change in Sumatra
Source: PLoS One. 2020 Aug 12;15(8):e0236144. doi: 10.1371/journal.pone.0236144 (PMC7423073; doi:10.1371/journal.pone.0236144)
Supplement: S1 Table — (DOCX) [file pone.0236144.s002.docx]

## **S1 Table. Small-medium sized wild cat detection encounter rate (#independent photo/100 trap days) and standard deviations in parentheses from the two survey periods and four study areas**

| **Study Area** | **Bungo** | | | | | | **Sipurak** | | | | | | **RKE** | | | | | | **Ipuh** | | | | | |
| --- | --- | --- | --- | --- | --- | --- | --- | --- | --- | --- | --- | --- | --- | --- | --- | --- | --- | --- | --- | --- | --- | --- | --- | --- |
| Feature | *#independent record* | | *% naïve occupancy* | | *RAI (SD)* | | *#independent record* | | *% naïve occupancy* | | *RAI (SD)* | | *#independent record* | | *% naïve occupancy* | | *RAI (SD)* | | *#independent record* | | *#% naïve occupancy* | | *RAI (SD)* | |
| ***Species records/ survey period*** | I | II | I | II | I | II | I | II | I | II | I | II | I | II | I | II | I | II | I | II | I | II | I | II |
| Clouded leopard | 18 | 33 | 29% | 24% | 0.53 (0.29) | 0.40 (0.66) | 10 | 21 | 19% | 14% | 0.98 (0.40) | 0.37 (1.19) | 12 | 62 | 13% | 40% | 0.58 (0.32) | 1.37 (2.29) | 20 | 35 | 33% | 21% | 1.03 (0.39) | 0.75 (1.79) |
| Golden cat | 6 | 18 | 10% | 17% | 0.79 (0.35) | 0.21 (0.69) | 5 | 38 | 10% | 30% | 0.33 (0.23) | 0.62 (1.39) | 5 | 26 | 13% | 25% | 0.63 (0.33) | 0.46 (0.97) | 7 | 17 | 14% | 16% | 1.20 (0.42) | 0.35 (1.11) |
| Leopard cat | 0 | 1 | 0% | 1% | 0 | 0.09 (0.28) | 0 | 0 | 0% | 0% | 0 | 0 | 0 | 0 | 0% | 0% | 0 | 0 | 0 | 1 | 0% | 1% | 0 | 0.03 (0.21) |
| Marbled cat | 6 | 5 | 10% | 6% | 0.26 (0.18) | 0.02 (0.15) | 15 | 21 | 29% | 17% | 0.33 (0.23) | 0.43 (0.88) | 13 | 23 | 22% | 9% | 0.24 (0.14) | 0.03 (0.16) | 23 | 9 | 35% | 11% | 0.36 (0.21) | 0.31 (0.21) |
