# Supplementary material for: Population dynamics of threatened felids in response to forest cover change in Sumatra
Source: PLoS One. 2020 Aug 12;15(8):e0236144. doi: 10.1371/journal.pone.0236144 (PMC7423073; doi:10.1371/journal.pone.0236144)
Supplement: S3 Table — (DOCX) [file pone.0236144.s004.docx]

## **S3 Table. Top occupancy models of golden cat from two periods of all study areas combined, of survey I and II, four study areas combined.**

| ***Golden cat*** | | | | | | | | | |
| --- | --- | --- | --- | --- | --- | --- | --- | --- | --- |
| ***Survey I*** | | | | | | | | | |
| No | Model | ѱ (+ SE) | *95% CIs* | p | K | AICc | ∆AICc | ModelLik | ModelWt |
| 1.1. | ѱ(forest), p(.) | 0.269 (0.021) | 0.150-0.408 | 0.31 | 3 | 273.706 | 0 | 1 | 23.18 |
| 1.2. | ѱ(forest+river+elev^2+def), p(.) | 0.269 (0.022) | 0.107-0.556 | 0.311 | 6 | 281.693 | 7.987 | 0.018 | 00.25 |
| 1.3. | ѱ(def), p(.) | 0.265 (0.009) | 0.153-0.416 | 0.315 | 3 | 284.649 | 10.943 | 0.004 | 00.05 |
| 1.4. | ѱ(.), p(.) | 0.265 (0.001) | 0.177-0.376 | 0.315 | 2 | 285.281 | 11.576 | 0.003 | 00.04 |
| 1.5. | ѱ(elev^2), p(.) | 0.265 (0.010) | 0.148-0.481 | 0.315 | 3 | 285.508 | 11.802 | 0.003 | 00.04 |
| 1.6. | ѱ(river), p(.) | 0.264 (0.005) | 0.155-0.421 | 0.315 | 3 | 286.497 | 12.791 | 0.002 | 00.02 |
|  |  |  |  |  |  |  |  |  |  |
| ***Survey II*** | | | | | | | | | |
| 2.1. | ѱ(elev^2), p(.) | 0.355 (0.009) | 0.196-0.577 | 0.144 | 3 | 365.904 | 0 | 1 | 07.09 |
| 2.2. | ѱ(.), p(.) | 0.355 (0.000) | 0.225-0.510 | 0.143 | 2 | 366.536 | 0.633 | 0.729 | 05.12 |
| 2.3. | ѱ(forest+river+elev^2+def), p(.) | 0.381 (0.015) | 0.140-0.681 | 0.137 | 6 | 366.669 | 0.765 | 0.682 | 04.52 |
| 2.4. | ѱ(def), p(.) | 0.360 (0.005) | 0.204-0.550 | 0.142 | 3 | 367.266 | 1.362 | 0.506 | 03.37 |
| 2.5. | ѱ(river), p(.) | 0.361 (0.005) | 0.198-0.557 | 0.142 | 3 | 367.53 | 1.626 | 0.444 | 03.10 |
| 2.6. | ѱ(forest), p(.) | 0.332 (0.001) | *0.219-0.471* | 0.227 | 3 | 469.256 | 103.352 | 0 | 0 |

Note: Model description i.e. ѱ(river), p(.) indicate occupancy with associated covariate distance to river and detection probability constant. *p* is detection probability, K is number of parameters, AICc is Akiake Information Criterion for small sample size, ∆AICc is difference between the best candidate and other plausible models, ModelLik is model likelihood of one model being selected as the best model and ModelWt is model weighting from the all models.
